# The GA4GH Workflow Execution Service: A Standard API for Workflow Execution and Management in Genomics

**DOI:** 10.64898/2026.10.20.760340

**Published:** 2026-10-24

**Authors:** Valentin Schneider-Lunitz, Landfried Kraatz, Patrick Magee, Venkat S. Malladi, Brian D. O’Connor, Susheel Varma, James Eddy, Hirotaka Suetake, Jeff Gentry, Michael R. Crusoe, Angel Pizarro, Junjun Zhang, Denis Yuen, Kyle Ellrott, Philip R. Kensche, Michael Lukowski, Reggan Thomas, Sasha V. Siegel, Alexander Kanitz, Tazro Ohta, Sven O. Twardziok

## Abstract

**Motivation:**

Rapid growth in life science data generation has led to large and increasingly distributed collections of sensitive genomic data. To analyse this data, researchers increasingly rely on computational workflows to enable scalable, portable and reproducible analysis. However, these workflows are executed by specialized workflow engines across heterogeneous compute environments, each with its own requirements. As a result, users often need engine- and compute-environment-specific knowledge to submit, monitor and manage workflow execution, increasing the barriers to interoperability. These challenges are compounded in large-scale projects, where workflow runs must be dispatched to local clusters, cloud platforms or data hosting infrastructures with different underlying technologies.

**Results:**

We describe the Global Alliance for Genomics and Health (GA4GH) Workflow Execution Service (WES) API, an approved GA4GH standard first released in 2018, together with its data model and the endpoints that define a unified interface for workflow execution. By standardizing workflow invocation, the WES API allows a single client to submit, monitor and cancel workflow runs across diverse workflow engines and compute backends, such as scheduling clusters or cloud platforms. We surveyed thirteen adoptions of the standard, covering standalone services and platforms in four programming languages. In a two-site demonstration, a WES implementation from Germany and one from Japan executed the same federated allele-frequency analysis with site-specific adjustments and only aggregated results leaving either site, demonstrating that it is possible to run the same analysis across independently built and operated WES services in different countries. From the gaps identified, we propose extensions for community discussion.

**Availability and implementations:**

The latest version of the WES API repository can be found at https://github.com/ga4gh/workflow-execution-service-schemas (DOI: https://doi.org/10.59756/m382x-ww ). The full Reference Documentation can be viewed at: https://ga4gh.github.io/workflow-execution-service-schemas/docs/

**Contact:**

valentin.schneider-lunitz@bih-charite.de

